# 肺腺癌合并血管内大B细胞淋巴瘤1例并文献复习

**DOI:** 10.3779/j.issn.1009-3419.2024.106.01

**Published:** 2024-02-20

**Authors:** Tongtong WANG, Xiaoyue CHEN, Guochen DUAN, Xiaopeng ZHANG, Qingtao ZHAO, Shun XU, Huanfen ZHAO

**Affiliations:** ^1^050000 石家庄，河北医科大学研究生学院（王彤彤）; ^1^Department of Graduate School, Hebei Medical University, Shijiazhuang 050000, China; ^2^050000 石家庄，河北省人民医院病理科（王彤彤，陈小悦，赵焕芬）; ^2^Department of Pathology, Hebei General Hospital, Shijiazhuang 050000, China; ^3^050000 石家庄，河北省儿童医院胸外科（段国辰）; ^3^Department of Thoracic Surgery, Hebei Children's Hospital, Shijiazhuang 050000, China; ^4^050000 石家庄，河北省人民医院胸外科（张霄鹏，赵庆涛）; ^4^Department of Thoracic Surgery, Hebei General Hospital, Shijiazhuang 050000, China; ^5^110001 沈阳，中国医科大学附属第一医院胸外科（许顺）; ^5^Department of Thoracic Surgery, The First Hospital of China Medical University, Shenyang 110001, China

**Keywords:** 肺肿瘤, 肺腺癌, 血管内大B细胞淋巴瘤, Lung neoplasms, Lung adenocarcinoma, Intravascular large B-cell lymphoma

## Abstract

血管内大B细胞淋巴瘤（intravascular large B-cell lymphoma, IVLBCL）是一种侵袭性结外大B细胞淋巴瘤，在同一器官与其他恶性肿瘤同时发生非常罕见，尤其是肺。本文报道1例肺腺癌合并IVLBCL的罕见病例。患者因腹泻伴发热、咳嗽入院。胸部计算机断层扫描（computed tomography, CT）示右肺上叶不规则斑片状高密度影，边缘有磨玻璃影。入院后给予患者抗感染等治疗，但仍有间断低热（最高37.5 ^o^C）。经皮肺穿刺活检（percutaneous lung biopsy, PLB）病理诊断为贴壁生长为主的腺癌，局部考虑浸润。手术后病理诊断为肺浸润性非黏液性腺癌合并IVLBCL。本文通过分析其临床病理特征，并复习相关文献，以提高临床和病理医师对该肿瘤的认识，避免漏诊或误诊。

## Abstract (en)

Intravascular large B-cell lymphoma (IVLBCL) is an aggressive extranodal large B-cell lymphoma, cocurrence in the same organ with other malignancies is very rare, especially in the lung. Here, we report a rare case of lung adenocarcinoma with IVLBCL. The patient was admitted to the hospital due to diarrhea associated with fever and cough. A computed tomography (CT) scan of the chest showed an irregular patchy high-density shadow in the upper lobe of the right lung with ground-glass opacity at the margin. After admission, the patient was given anti-infection treatment, but still had intermittent low fever (up to 37.5 ^o^C). The pathological diagnosis of percutaneous lung biopsy (PLB) was lepidic-predominant adenocarcinoma with local infiltration, which was proved to be invasive nonmucinous adenocarcinoma of the lung with IVLBCL after surgery. This paper analyzed the clinicopathological characteristics and reviewed the relevant literature to improve the knowledge of clinicians and pathologists and avoid missed diagnosis or misdiagnosis.

血管内大B细胞淋巴瘤（intravascular large B-cell lymphoma, IVLBCL）是一种非常罕见的侵袭性结外大B细胞淋巴瘤，以淋巴瘤细胞选择性在小血管腔内，特别是毛细血管腔内生长为特征。其不良预后的一个原因是由于临床表现和影像学检查均无特异性，导致诊断延迟。肺IVLBCL罕见，诊断时应与脉管内癌栓、微乳头状腺癌及血管内自然杀伤/T细胞淋巴瘤（intravascular natural killer/T cell lymphoma, IVNKTL）相鉴别。本文报道1例肺腺癌合并IVLBCL的罕见病例，并进行文献复习，旨在探讨其临床病理特征及经验，为疾病的诊疗及预后提供依据。

## 1 病例资料

患者男性，66岁，因腹泻，伴发热、咳嗽10天，当地诊所抗感染治疗无效，于2021年9月30日入院。入院诊断：腹泻待查。查体：无阳性体征。胸部计算机断层扫描（computed tomography, CT）示：右肺上叶后段见不规则斑片状高密度影（图1A），可见斑点状钙化，边缘见少许磨玻璃影，双侧少量胸腔积液；脾大。实验室检查中肿瘤标志物：甲胎蛋白为2.05 ng/mL、癌胚抗原为1.5 ng/mL、糖类抗原199为5.16 U/mL、糖类抗原125为56.22 U/mL（升高）、糖类抗原153为17.96 U/mL、铁蛋白为1034 ng/mL（升高）、总前列腺特异性抗原为1.02 ng/mL、鳞状上皮细胞癌抗原为1.118 ng/mL。其他指标：乳酸脱氢酶为471.5 U/L（升高）、D-二聚体为2.6 mg/L（升高）、C反应蛋白为37.56 mg/L（升高）、红细胞沉降率为38 mm/h（升高）；白蛋白为27.9 g/L（降低）、淋巴细胞计数为0.82×10^9^/L（降低）。

**图1:**
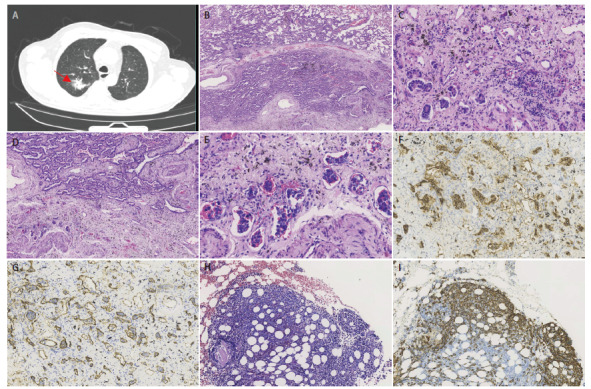
患者影像学表现及活检结果。A：胸部CT示右肺上叶后段可见不规则斑片状高密度影（↑），可见斑点状钙化，边缘可见少许磨玻璃影；B：附壁状腺癌及腺泡状腺癌（HE染色，×40）；C：微乳头状腺癌附近小血管腔内见异型大细胞（HE染色，×300）；D：腺泡状腺癌周围小血管腔内见异型大细胞（HE染色，×100）；E：小血管腔内的异型大细胞，细胞核大，深染/空泡状，可见核仁（HE染色，×400）；F：小血管腔内异型大细胞CD20阳性（SP法，×200）；G：小血管内皮细胞CD34阳性（SP法，×200）；H：骨髓组织内异型大淋巴细胞（HE染色，×200）；I：骨髓组织内异型大淋巴细胞CD20阳性（SP法，×200）。

入院后给予调节肠道菌群、止泻及抗感染等治疗，治疗5 d后大便正常，复查C反应蛋白为2.95 mg/L、红细胞沉降率为12 mm/h，均降至正常，但患者仍有间断低热（最高37.5 ^o^C）。为查明发热原因，进一步查结核感染T细胞检测、结核菌素试验、EB病毒（Epstein-Barr virus, EBV）聚合酶链反应（polymerase chain reaction, PCR）检测、巨细胞病毒PCR检测等均为阴性。

结合患者胸部CT及实验室检查，考虑恶性肿瘤可能性大，于2021年10月14日行CT引导下经皮肺穿刺活检术，肉眼为灰白色穿刺组织4条，长共1.5 cm，直径0.1 cm，病理诊断为以附壁状生长为主的腺癌，局部考虑浸润，建议完整切除后进一步确定。遂于2021年10月18日行胸腔镜下右肺上叶切除术+淋巴结清扫术。送检右肺上叶及淋巴结6组切除标本，肺叶大小为14 cm×9 cm×3.5 cm，部分支气管长2 cm，周径4 cm，距支气管断端4 cm，紧邻胸膜见一肿物，大小为2.5 cm×2.3 cm×2 cm，切面灰白质硬。镜下观察：肿瘤周边区域为典型附壁状腺癌，中央区域纤维化背景中见典型腺泡状腺癌和微乳头状腺癌（图1B、1C），其间小血管腔内见红细胞和多少不等的异型大细胞，异型大细胞黏附性差，散在血管腔内，细胞核大，不规则，深染或空泡状，可见核仁，核分裂象可见（图1D、1E），间质内未见散在形态类似的肿瘤细胞。免疫组化染色：（1）腺癌表达甲状腺转录因子1（thyroid transcription factor-1, TTF-1）、天冬氨酸蛋白酶A（Napsin A）、细胞角蛋白7（cytokeratin 7, CK7），增殖细胞核抗原（Ki-67）阳性率约3%，其中微乳头状腺癌上皮细胞膜抗原（epithelial membrane antigen, EMA）阳性，显示极性翻转；（2）小血管腔内异型大细胞表达CD20（图1F）、CD79α，多发性骨髓瘤癌基因蛋白1（multiple myeloma oncogene 1, MUM1）（60%阳性），Bcl-6（60%阳性），Bcl-2（70%阳性），C-MYC（50%阳性），Ki-67阳性率90%；不表达细胞角蛋白（pan-cytokeratin, CKpan）、CD3、CD10、间变性淋巴瘤激酶（anaplastic lymphoma kinase, ALK）；（3）血管内皮细胞CD34阳性（图1G）和D2-40阴性证实异型大细胞位于血管腔内。原位杂交检测：EB病毒编码小RNA（EBV encoded RNA, EBER）阴性。病理诊断：（1）（右肺上叶）浸润性非黏液性腺癌（附壁状55%，腺泡状40%，微乳头状5%），合并血管内大B细胞淋巴瘤；（2）支气管断端未见肿瘤，未见神经浸润及胸膜侵犯；（3）区域淋巴结15枚未见癌转移。

病理诊断完成后，临床修正诊断为：（1）（右肺上叶）浸润性非黏液性腺癌（T1cN0M0，IA3期）；（2）血管内大B细胞淋巴瘤（IV期，高危）；（3）急性肠炎。对于切缘阴性的IA期肺癌患者，根据中华医学会肺癌临床诊疗指南（2023版），无需术后辅助治疗，定期随访即可。根据美国国立综合癌症网络（National Comprehensive Cancer Network, NCCN）的弥漫大B细胞淋巴瘤（diffuse large B-cell lymphoma, DLBCL）实践指南（2023.V6版），IV期IVLBCL首选R-CHOP（利妥昔单抗+环磷酰胺+多柔比星+长春地辛+泼尼松）方案治疗。患者治疗1个疗程后，2021年12月1日正电子发射计算机断层扫描（positron emission tomography/CT, PET/CT）显示：双肺未见氟脱氧葡萄糖（fluorodeoxyglucose, FDG）代谢异常（Deauville评分: 1分）；右髂前上棘局部髓腔内FDG代谢增高灶；脾大。第二疗程化疗前及化疗后各行骨髓穿刺活检1次，结果均阴性，考虑为化疗后骨髓应激性改变。患者接受R-CHOP方案治疗3个疗程后，乳酸脱氢酶降至正常（231.9 U/L），提示治疗有效；继续治疗5个疗程后发热、咳嗽等症状消失，复查全身CT未见明显异常，遂停止治疗。定期复查至2023年6月（停止治疗后14个月），患者再次出现发热症状，骨髓穿刺活检证实IVLBCL侵犯骨髓（图1H、1I），患者继续接受R-CHOP方案治疗1个疗程后，行骨髓穿刺活检，结果为阴性。目前仍规律化疗中。

## 2 讨论

### 2.1 概述及文献复习

第5版世界卫生组织（World Health Organization, WHO）肺肿瘤分类中，肺腺癌包括微浸润性腺癌、浸润性非黏液性腺癌、浸润性黏液腺癌、胶样腺癌、胎儿型腺癌和肠型腺癌。其中浸润性非黏液性腺癌是肺腺癌的最常见类型，包括附壁状、腺泡状、乳头状、微乳头状和实体状腺癌，通常多种形态同时存在。本例的浸润性非黏液性腺癌成分包含了附壁状腺癌、腺泡状腺癌和微乳头状腺癌，组织形态典型，本文不做更多讨论。

早在第4版WHO肺肿瘤分类中，淋巴组织肿瘤章节已列出IVLBCL。IVLBCL是一种非常罕见的侵袭性结外大B细胞淋巴瘤，它的特征是在小血管，特别是毛细血管内存在淋巴瘤细胞。肺原发IVLBCL罕见，迄今国内外文献报道57例，加上本例共58例（表1）。58例患者中，年龄32-84岁，男女比例为1.4:1。临床表现包括呼吸困难、咳嗽、胸腔积液、发热、体重减轻等，亦有以肺动脉高压为初始症状^[1]^的报道。诊断方式包括经支气管肺活检（transbronchial lung biopsy, TBLB）、外科肺活检、胸腔镜肺活检（thoracoscopic lung biopsy, TLB）等。PET/CT检查阳性率为78.6%（11/14）。治疗方面，接受CHOP（环磷酰胺+多柔比星+长春地辛+泼尼松）或R-CHOP方案的患者中，缓解率为74.3%，完全缓解率为22.9%。

**表1:** 58例肺IVLBCL病例临床特征分析

Item	Clinical characteristics
Gender	Male-female ratio: 1.4 to 1
Age	The mean age was 61 yr (32-84), male was 63 yr and female was 59 yr.
Initial symptoms	Respiratory symptoms were predominant (47 cases), including dyspnea, cough, and pleural effusion. Secondly, B symptoms, including fever (42 cases) and weight loss (14 cases). Rare symptoms were pulmonary hypertension (2 cases) and pulmonary embolism (1 case).
Diagnostic methods	Transbronchial lung biopsy (23 cases), thoracoscopic lung biopsy (10 cases), surgical lung biopsy (7 cases), transbronchial frozen biopsy (3 cases), percutaneous lung biopsy (3 cases), autopsy (2 cases), skin biopsy (2 cases), lymph node biopsy (1 case), pulmonary microvascular cytology (1 case), not mentioned (6 cases).
PET/CT	There were 14 cases, including 11 cases with metabolic abnormalities, with a positive rate of 78.6%.
Treatment	A total of 42 patients were treated with chemotherapy, and among them, 35 patients received the CHOP or R-CHOP therapeutic regimen, with 18 cases remission, 8 cases complete remission, 2 cases relapsed, 7 cases died.

IVLBCL: intravascular large B-cell lymphoma; PET: positron emission tomography; CHOP: cyclophosphamide, vindesine, adriamycin and prednisone; R-CHOP: rituximab, cyclophosphamide, vindesine, adriamycin and prednisone.

肺腺癌合并IVLBCL的情况极为罕见，迄今国内外文献^[2]^仅见1例报道，是Satoh等在2019年报道的1例73岁男性患者，因严重呼吸困难入院。胸部CT示右肺下叶实性病变，双肺多发斑片状磨玻璃影；PET/CT示双肺FDG代谢异常；实验室检查：可溶性白细胞介素-2受体（2098 U/L）、乳酸脱氢酶（645 U/L）均升高。术后病理诊断为肺浸润性腺癌合并IVLBCL，伴骨髓转移。接受R-CHOP治疗6个疗程后，患者症状消失，复查胸部CT显示双肺磨玻璃影消失；PET/CT显示肺对FDG的异常摄取消失。本文病例以发热、咳嗽为主要症状就诊，影像和实验室检查与文献报道有相同之处，患者接受R-CHOP方案治疗8个疗程后症状消失，复查全身CT未见明显异常，但停止治疗后14个月，患者再次出现发热症状，骨髓穿刺活检证实IVLBCL侵犯骨髓，继续接受R-CHOP方案治疗1个疗程后，行骨髓穿刺活检，结果为阴性。

IVLBCL可扩散到全身的多个器官^[3]^，包括中枢神经系统、骨髓、肝、脾、肾、肺和皮肤等，累及淋巴结较少见。肺IVLBCL通常被误诊为间质性肺炎，一方面，是由于其临床表现的非特异性，另一方面，IVLBCL一般不形成血管外肿瘤/肿块^[3]^，这给诊断造成了极大的困难。肺IVLBCL在CT上可表现为放射状或弥漫性磨玻璃影，高密度或实性斑片影和结节，也可见小叶间隔增厚和肺炎，极少数病例中可见反晕征^[4]^；在PET/CT上可呈现“热肺”征^[5]^。胸部CT及PET/CT可用于肺部疾病的筛查和诊断，但最终诊断仍然取决于病理活检。目前，最常用的活检方式是TBLB，其次是外科肺活检和TLB。经支气管冷冻活检是最近可用于诊断肺IVLBCL的新方法^[6]^，既可获得更大的样本，损伤也比手术活检小。当患者存在凝血障碍等对手术不耐受的情况时，可选择经支气管冷冻活检。本例患者因腹泻、发热、咳嗽入院，查胸部CT发现病变，最终经术后病理确诊。

组织病理学上，肿瘤淋巴细胞通常局限在小动脉、静脉和毛细血管的管腔内，有时可见纤维蛋白血栓。肿瘤细胞体积大，细胞核呈囊泡状，核仁突出，核分裂常见。肿瘤很少是间变性的。免疫组化染色，肿瘤细胞通常呈B细胞阳性（CD20和CD79α），常见CD5表达。遗传学方面，大多数病例显示免疫球蛋白基因重排。在染色体1、6和18中发现了结构畸变，特别是1p和18三体。文献^[7]^报道，在亚洲国家的IVLBCL患者中发现了6q13、8p11和19q13等突变以及4号和8号染色体的丢失。本例在肺腺癌背景中间质小血管腔内见红细胞和多少不等的异型大细胞，异型大细胞黏附性差，散在血管腔内，细胞核大，不规则，深染或空泡状，可见核仁，核分裂象可见。本例免疫组化染色，异型大细胞CD20和CD79α阳性证实为大B细胞淋巴瘤；小血管CD34阳性和D2-40阴性证实位于血管腔内。

### 2.2 鉴别诊断

由于IVLBCL在临床表现及影像学检查均无特异性，因此需要更加注重病理检查及免疫组织化学方法的检测。（1）IVNKTL：罕见情况下NK/T细胞淋巴瘤可能显示血管内受累。IVNKTL与IVLBCL均为毛细血管腔内异型淋巴细胞，免疫组化染色IVNKTL与IVLBCL分别表达T细胞标志物和B细胞标志物可鉴别，同时，IVNKTL大多EBER及CD56阳性，常表达一种或多种细胞毒性标志物（如TIA-1、颗粒酶B和穿孔素），而IVLBCL则EBER及CD56阴性，不表达细胞毒性标志物^[8]^，也有助于鉴别。（2）脉管内癌栓：合并肺腺癌的病例需鉴别。IVLBCL细胞黏附性差，结合免疫组化染色（CD20和CD79α阳性，上皮标记阴性）可鉴别。（3）微乳头状腺癌：合并肺腺癌的病例需鉴别。微乳头状腺癌细胞有黏附性，癌巢周围腔隙无内皮细胞衬附，结合免疫组化染色（CD20和CD79α阴性，上皮标记阳性）可鉴别。

### 2.3 治疗和预后

根据中华医学会肺癌临床诊疗指南（2023版），早期肺癌首选根治性外科手术切除，对于切缘阴性的IA期肺癌患者，无需术后辅助治疗，定期随访即可。根据NCCN的DLBCL实践指南（2023.V6版），目前推荐的一线治疗方案仍然是经典的R-CHOP方案，并在全部治疗完成后评估疗效，如达到完全缓解，则可以定期复查。对于年轻低危患者，6个疗程的R-CHOP方案是年轻预后良好的DLBCL患者最佳的治疗方案之一；对于60-80岁、无心功能不全的患者，8个疗程的R-CHOP与6个疗程的R-CHOP疗效相当^[9]^。既往IVLBCL预后一直很差，目前使用CHOP或R-CHOP方案治疗成功的病例已有报道（表1）。文献报道58例肺IVLBCL患者中，42例接受化疗，其中35例采用CHOP或R-CHOP方案治疗，18例缓解，8例完全缓解，2例复发，7例死亡，缓解率为74.3%，完全缓解率为22.9%。有报道^[10]^显示，预后营养指数（prognostic nutritional index, PNI）联合D-二聚体对初诊DLBCL患者预后有提示作用，即高PNI（≥44.775）和低D-二聚体（<0.835）的DLBCL患者具有更好的预后，据此本例患者非高PNI（32）和低D-二聚体（2.6），接受R-CHOP方案治疗8个疗程后症状消失，全身CT未见明显异常，停止治疗后14个月，再次出现发热症状，骨髓穿刺活检证实IVLBCL骨髓侵犯。目前规律化疗中。

综上所述，IVLBCL是一种非常罕见的侵袭性结外大B细胞淋巴瘤，预后差，死亡率高。临床表现及影像学检查均无特异性，诊断困难。过去常常在尸检时才明确诊断，以致于错过了治疗机会。当患者出现不明原因发热、咳嗽等症状时，胸部CT显示磨玻璃影、反晕征，PET/CT显示“热肺征”，肿瘤标志物及乳酸脱氢酶、可溶性白细胞介素-2受体、D-二聚体等升高时，应考虑IVLBCL的可能，及时做病理学活检明确诊断。因此，综合分析临床表现、实验室检查和影像学特征，及时进行病理学活检，是IVLBCL早发现、早治疗的必要手段。

**Competing interests**

The authors declare that they have no competing interests.
